# A Practical Treatise on the Venereal Disease; Founded on Six Lectures on That Subject, Delivered in the Session of 1838-9, at the Aldersgate-Street School of Medicine

**Published:** 1841-04

**Authors:** 


ART. IV.—*A Practical Treatise on the Venereal Disease; founded on Six Lectures on that subject, delivered in the Session of* 1838-9, *at the Aldersgate-street School of Medicine. With Plates.* By F. C. SKEY, F.R.S.—*London*, 1841. 8vo, pp. 195.

WE are in some measure at a loss to explain Mr. Skey's motive in giving to the medical profession at the present day the volume under notice. It certainly is not, as its title would lead us to expect, a *treatise* on the venereal disease, but an imperfect monogragh on some of its primary and secondary forms. The principal object of Mr. Skey's work is to advocate the simple or non-mercurial treatment of primary venereal sores, with the exception of the indurated primary chancre. Mr. Skey comes forward at the eleventh hour as the advocate of a system which has been gaining ground in Europe for the last five and twenty years; upon which hundreds of volumes have been written; and with which all medical men are or ought to be familiar. In limiting the treatment by mercury to the indurated primary sore, Mr. Skey merely recapitulates the rules laid down by Mr. Carmichael, and the foreign writers generally.

The first chapter of the work contains a short historical account of the origin and progress of venereal diseases; and here Mr. Skey speaks of the spontaneous or self-generated origin of syphilis, in which "the elements of the poison lie dormant, and may be developed by the action of a simple irritant." We lament that Mr. Skey, when speaking of the mode of propagation of venereal disease, has omitted all mention of the data which are furnished by inoculation, and the use of the speculum. The latter instrument has shown that primary venereal sores and gonorrhœal discharges are as frequently seated deep in the vagina, on the neck of the uterus, and even in the os uteri, as they are upon the external parts; and hence any opinions on the propagation of primary sores or gonorrhœa, furnished on the examination of the external parts only, must be received with much reservation. Mr. Skey says, "Do not venereal sores almost invariably occupy the external organs?" Most certainly they do not. By neglecting the information furnished from these two sources Mr. Skey would again plunge his readers into that confusion from which the history of the propagation of syphilis had been in some degree rescued by the means in question.

We find nothing to detain us in the second, third, fourth, and fifth chapters. They however contain some valuable cases illustrating the positions already laid down, and which we have said are those of generally well-informed medical men of the present day, at least where Mr. Hunter's dogmas have not been too blindly worshipped. The last chapter is devoted to gonorrhœa, its varieties and treatment. Here the doctrine of spontaneous origin is again broached. On many of the points contained in this chapter we are directly opposed to Mr. Skey's views. We certainly believe that the urethral discharges produced by sexual intercourse are of various kinds, but we also believe the true gonorrhœa virulenta to be a specific disease, capable of propagation under the same forms, and very easily to be distinguished from those discharges we may term simple, which are due to cohabitation with females suffering from leucorrhœa, or various inflamed and irritable conditions of the vagina. We are sorry our limits will not permit us to examine Mr. Skey's views in this matter more closely. Besides the cases already referred to, there are many other very instructive ones scattered through the work; we would, however, remark that many of these cases cannot be implicitly relied on, being founded only on the examination of the external parts.

